# Papillary Thyroid Microcarcinoma: A Nomogram Based on Clinical and Ultrasound Features to Improve the Prediction of Lymph Node Metastases in the Central Compartment

**DOI:** 10.3389/fendo.2021.770824

**Published:** 2022-01-12

**Authors:** Jing Ye, Jia-Wei Feng, Wan-Xiao Wu, Jun Hu, Li-Zhao Hong, An-Cheng Qin, Wei-Hai Shi, Yong Jiang

**Affiliations:** ^1^ Department of Thyroid Surgery, The Third Affiliated Hospital of Soochow University, Changzhou First People’s Hospital, Changzhou, China; ^2^ The Affiliated Suzhou Hospital of Nanjing Medical University, Suzhou Municipal Hospital, Suzhou, China; ^3^ The Affiliated Hospital of Nanjing Medical University, Changzhou Second People’s Hospital, Changzhou, China

**Keywords:** thyroid cancer, microcarcinoma, nomogram, central lymph node metastases, recurrence-free survival

## Abstract

**Background:**

Accurate preoperative identification of central lymph node metastasis (CLNM) is essential for surgical protocol establishment for patients with papillary thyroid microcarcinoma (PTMC). We aimed to develop a clinical and ultrasound characteristics-based nomogram for predicting CLNM.

**Methods:**

Our study included 399 patients who were pathologically diagnosed with PTMC between January 2011 and June 2018. Clinical and ultrasound features were collected for univariate and multivariate analyses to determine risk factors of CLNM. A nomogram comprising the prognostic model to predict the CLNM was established, and internal validation in the cohort was performed. The Cox regression model was used to determine the risk factors for recurrence-free survival (RFS) and cumulative hazard was calculated to predict prognosis.

**Results:**

Three variables of clinical and US features as potential predictors including sex (odd ratio [OR] = 1.888, 95% confidence interval [CI], 1.160-3.075; *P* =0.011), tumor size (OR = 1.933, 95% CI, 1.250-2.990; *P* =0.003) and ETE (OR = 6.829, 95% CI, 3.250-14.350; *P <*0.001) were taken into account. The predictive nomogram was established by involving all the factors above used for preoperative prediction of CLNM in patients with PTMC. The nomogram showed excellent calibration in predicting CLNM, with area under curves (AUC) of 0.684 (95% CI, 0.635 to 0.774). Furthermore, tumor size, multifocality, presence of ETE, vascular invasion, and CLNM were the significant factors related to the RFS.

**Conclusion:**

Through this easy-to-use nomogram by combining clinical and US risk factor, the possibility of CLNM can be objectively quantified preoperatively. This prediction model may serve as a useful clinical tool to help clinicians determine an individual’s risk of CLNM in PTMC, thus make individualized treatment plans accordingly.

## Introduction

Papillary thyroid carcinoma (PTC) has become one of the most common malignancies of the endocrine system, with an average annual incidence growth rate of 6% worldwide (1). If the maximum size of PTC is ≤ 1.0 cm, it is defined as papillary thyroid microcarcinoma (PTMC) by the World Health Organization (2). Although most thyroid cancer usually grows slowly and has a good prognosis, some PTMC tend to metastasize to regional lymph nodes (3). As previous reported (4–6), the incidence of central lymph node metastasis (CLNM) was the highest among the neck compartment, ranging from 19% to 76%. Once diagnosed with CLNM, especially with large macroscopic lymph node metastases, patients were at high risk for poor prognosis and regional recurrence (7).

With the application of routine ultrasonography (US) and the necessary fine-needle aspiration biopsy (FNAB), the diagnostic rate of PTMC has been greatly improved (8). However, the preoperative detection rate of CLNM still remains quite low due to the limitations of current imaging technology (9). A recent meta-analysis showed that the sensitivity of preoperative US to diagnose CLNM of PTC was only 0.33 (10). Considering the high prevalence of CLNM, prophylactic central lymph node dissection (CLND) is usually performed in Asian countries such as Japan and China. As a result, this may cause more complications after thyroid surgery, such as recurrent laryngeal nerve injury, hypoparathyroidism, and chyle leakage (11–14). Hence, an effective and noninvasive tool that could evaluate the invasion of tumors and determine the extent of operation before surgery may help to develop the best treatment plan for PTMC patients.

Unlike other researchers who simply identified the risk factors for CLNM (3), we developed a clinical and US characteristics-based nomogram to evaluate the probability of CLNM in patients with PTMC. With our accurate and convenient evaluation system, clinicians would be able to quantify the possibility of CLNM preoperatively and make better clinical decisions. Moreover, risk factors of recurrence-free survival (RFS) and cumulative hazards were calculated for individualized treatment plan formulation and patient outcomes would be improved eventually.

## Materials and Methods

### Patient Recruitment

This research was performed according to the Declaration of Helsinki. The multi-center retrospective cohort research was approved by the Ethical Committee of the Third Affiliated Hospital of Soochow University and Suzhou Municipal Hospital. All patients provided written informed consent and agreed to use their clinical data for this study. From January 2011 to June 2018, all pathologically proven PTMC cases who were treated at this two hospitals were reviewed retrospectively. In our research, the inclusion criteria were: (1) All patients received surgery for the first time; (2) The primary lesion was located in the thyroid gland; (3) No family history of thyroid cancer; (4) No other malignant tumors; (5) No head and neck radiation exposure history; (6) No distant metastasis; (7) Postoperative pathology confirmed PTC; (8) Complete clinical data. The exclusion criteria were: (1) patients who also had other types of TC; (2) patients who did not undergo radical surgery or CLND; (3) reoperation; (4) diagnosed tumors of other organs previously; (5) history of neck radiation exposure or hereditary malignancy; (6) clinically and/or pathologically detected distant metastasis; (7) patients who were lost to follow-up midway or with incomplete clinical information. In the end, we included and assessed a total of 399 patients in the research.

### Preoperative Examination and Surgical Procedures

A thorough examination would be conducted once a thyroid nodule was discovered. In addition to routine testing of serum thyroid hormones, neck US was usually carried out to assess the characteristics of thyroid nodules and lymph nodes in the central compartment. Moreover, FNAB was performed preoperatively to diagnose benign or malignant nodules and pathological types. The BRAF V600E mutation was only conducted since 2017. In our research, the preoperative US variables of thyroid nodules mainly included: tumor size (the largest dimension of primary tumor), multifocality (two or more PTC lesions), and extrathyroidal extension (ETE).

Considering FNAB and imaging findings, if PTMC patients were in any of the following conditions (isthmus lesion, bilateral lesions, or presence of gross ETE), a total thyroidectomy (TT) with bilateral CLND would be performed. Otherwise, only lobectomy with CLND on the affected side would be performed (2). The central compartment (CC) means level VI of neck lymph node partition. The histological analysis of all surgical specimens was routinely conducted in the department of pathology.

### Histopathological Examination

Pathological sections and further immunohistochemistry of the specimens were performed by at least two experienced pathologists. Vascular invasion was confirmed by pathologists through the microscope. Disease recurrence was categorized as local (regional cervical lymph nodes or thyroid bed) or distant (other organs) and confirmed by cyto-histopathology or imaging. RFS was identified as the length of time between the date of completion of the thyroid surgery and dates of recurrence. RFS and cumulative hazards were used to evaluate the outcomes.

### Postoperative Strategies and Follow-up

The postoperative patients were treated with levothyroxine suppression therapy. Serum thyroglobulin (Tg) and Tg antibodies, US scan of the neck area and physical examinations were conducted every 3 to 6 months for high-risk patients and every 6 to 12 months for middle- and low-risk patients according to the 2015 ATA guidelines. When detected serum Tg and/or Tg antibody levels were significantly elevated, imaging examinations like CT or MRI or further histological confirmation would be performed. Follow-up information was acquired through telephone contact or outpatient consultations.

### Statistical Analysis

Clinical data analysis in our study was conducted by utilizing software SPSS for Windows (version 25.0) and the R software (version 3.6.0). Categorical data were presented as numbers and percentages, while continuous data were presented as means ± standard deviations. Pearson’s chi-square test or Fisher’s exact test was applied for categorical data depending on the situation. Univariate analysis of CLNM-related risk factors in PTMC patients was also performed by using Pearson’s chi-square test or Fisher’s exact test. The statistically significant variables in the univariate analysis were further included in the multivariate logistic regression analysis to identify independent risk factors of CLNM. Next, the rms/pROC package in the R software was used to construct a predictive nomogram of risk factors based on the results of multivariate logistic regression, which could convert each variable coefficient into a score from 0 to 100. The receiver operating characteristic (ROC) curve was employed to quantify and compare the ability of predicted cases and observed cases to predict CLNM in PTMC. The R software was also used to evaluate the accuracy of the best cut-off value. In order to assess the performance of the nomogram, a calibration chart containing 1,000 bootstrap samples was further performed to compare the predicted probabilities with the actual probabilities of CLNM. Univariate Cox regression analysis was used to identify the risk factors of RFS. The Kaplan-Meier method and the Log rank test were performed to calculate cumulative hazard to assess the differences between groups.

## Results

### Base Clinical and US Features of PTMC Patients

The clinical data of 399 patients with PTMC were collected in our study. 103 (25.8%) men and 296 (74.2%) women at a mean age of 46.0 ± 11.3 years (range 21-77 years) were included in our study. According to our US findings, the average maximum tumor diameter was 0.60 ± 0.25 cm; 212 (53.1%) were larger than 0.5 cm and 187 (46.9%) were 0.5 cm or smaller. One hundred and four (26.1%) patients had multifocal lesions. ETE were detected in 49 (12.3%) patients. Among 39 patients undergoing BRAF mutation analysis, 34 (87.2%) were positive for the gene mutation. Vascular invasion was evident in 12 (3.0%) cases.

In our study, all 399 PTMC patients underwent CLND, of which 156 (39.1%) patients were detected with CLNM after surgery. Pathology after surgery confirmed that the mean number of central lymph nodes resected was 5.8 ± 4.6 (range 2-26), while the mean number of metastases was 2.3 ± 1.2 (range 0-13). According to postoperative pathology, the average size of positive central lymph node was 0.6 ± 0.3 cm. The post-operative median follow-up was 25 months (range 3-89 months). During the follow-up, 18 patients (4.5%) developed recurrent disease, including 12 patients (3.0%) who had cervical lymph node recurrence, 3 patients (0.7%) who had contralateral lobe recurrence, 2 patients (0.5%) who had both lymph node and contralateral lobe recurrence and 1 patient (0.3%) who had lung recurrence.

### Correlation Between Clinical and US Features and CLNM in PTMC Patients

In 399 PTMC cases, clinical variables that significantly related to CLNM were age, sex, tumor size, ETE and vascular invasion. In our univariate analysis, compared with non-CLNM, the incidence of CLNM in patients with age <55 years, male, tumor size >0.5 cm, ETE, and vascular invasion was substantially higher (*P*=0.027, *P*=0.001, *P*=0.001, *P*<0.001, and *P*=0.010, respectively). Further, the multivariate analysis indicated that male (OR: 1.888, 95% CI: 1.160-3.075, *P*=0.011), tumor size >0.5 cm (OR: 1.933, 95% CI: 1.250-2.990, *P*=0.003) and ETE (OR: 6.829, 95% CI: 3.250-14.350, *P*<0.001) were independent predictive factors associated with CLNM in PTMC (**Table 1**).

**Table 1:** Univariate and multivariate analyses of factors associated with CLNM and score in patients with PTMC.

Characteristics	CLNM, No. (%)			Multivariate analysis	*P* value	Score
	Presence (n = 156)	Absence (n = 243)	*P* value	Adjusted OR (95% CI)		
Age (Y)						
≥55	69 (44.2%)	135 (55.6%)		1		
<55	87 (55.8%)	108 (44.4%)	0.027	1.408 (0.911-2.177)	0.123	
Sex						
Female	102 (65.4%)	194 (79.8%)		1		0
Male	54 (34.6%)	49 (20.2%)	0.001	1.888 (1.160-3.075)	0.011	33
Tumor size (cm)						
≤0.5	57 (36.5%)	130 (53.5%)		1		0
>0.5	99 (63.5%)	113 (46.5%)	0.001	1.933 (1.250-2.990)	0.003	34
Multifocality						
Absence	107 (68.6%)	188 (77.4%)				
Presence	49 (31.4%)	55 (22.6%)	0.051			
ETE						
Absence	117 (75.0%)	233 (95.9%)		1		0
Presence	39 (25.0%)	10 (4.1%)	<0.001	6.829 (3.250-14.350)	<0.001	100
Vascular invasion						
Absence	147 (94.2%)	240 (98.8%)		1		
Presence	9 (5.8%)	3 (1.2%)	0.010	1.092 (0.201-5.923)	0.919	
BRAF V600E mutation^*^						
Negative	2 (10.5%)	3 (15.0%)				
Positive	17 (89.5%)	17 (85.0%)	0.676			

Y, year; SD, standard deviation; ETE, extrathyroidal extension; CLNM, central lymph node metastasis.

* BRAF mutation analysis was started in 2017 and it was performed in 39 patients with PTMC.

### Development and Performance of the Nomogram

**Figure 1:**
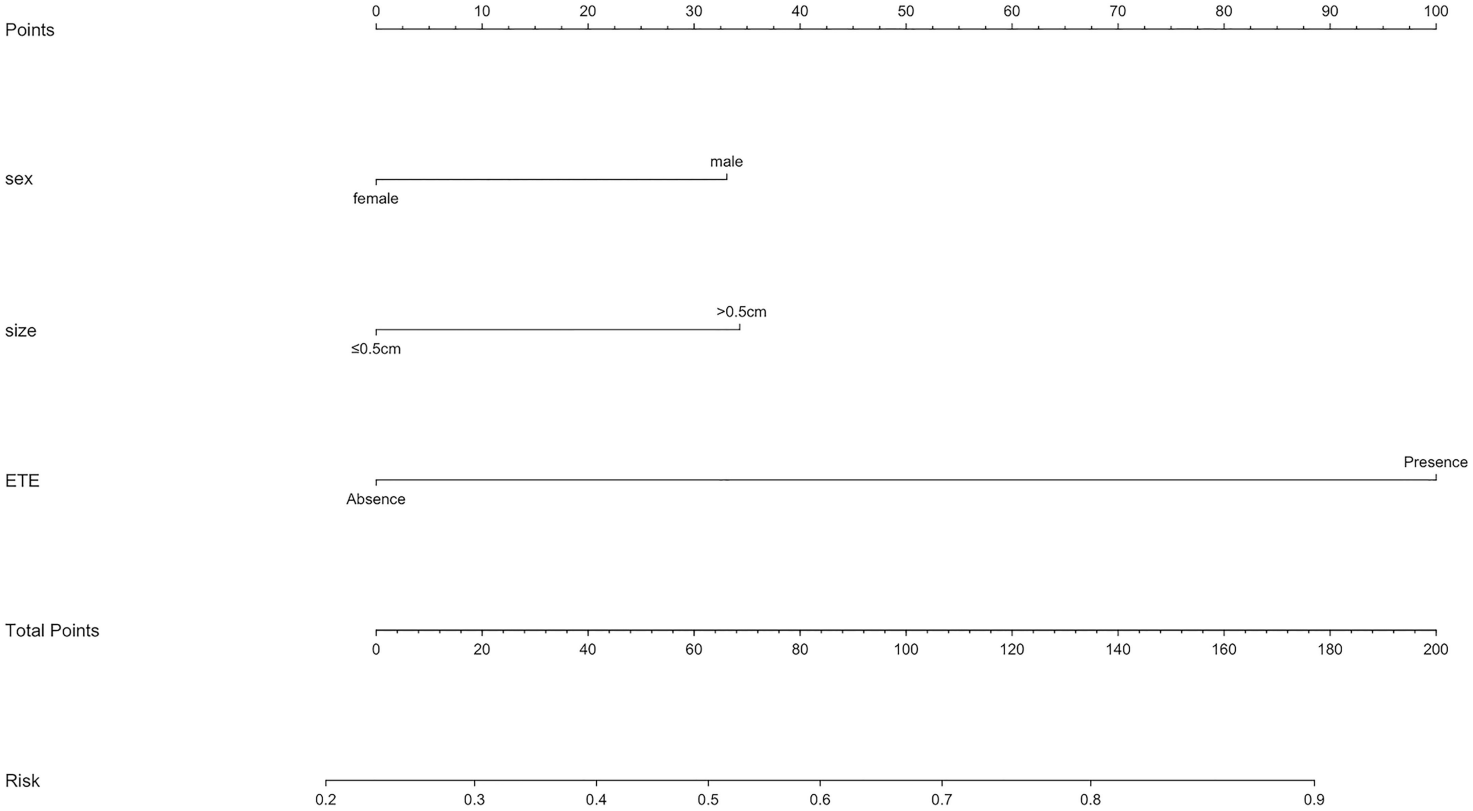
Nomogram for predicting CLNM in patients with PTMC.

**Figure 2:**
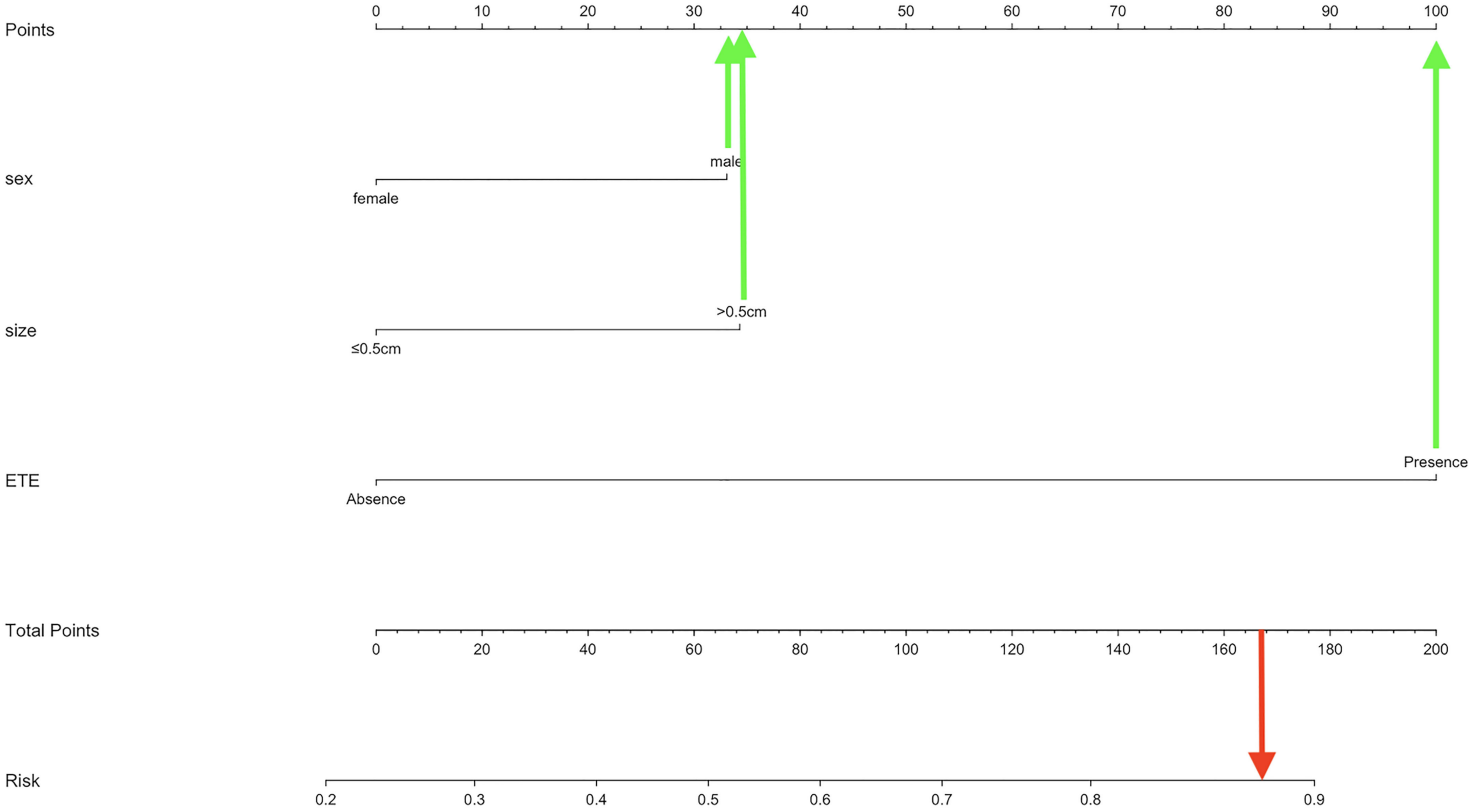
Demonstration of the usage of our nomogram.

As mentioned above, a nomogram was created from previous independent risk factors (sex, tumor size, and ETE) to assess the CLNM incidence of PTMC preoperatively (**Figure 1**). According to the analysis, ETE contributes the most to the prediction model, followed by tumor size. These variables were finally rated on a scale of 0-100, and the risk rate of CLNM would be calculated by summing up all total scores. The usage of nomogram (**Figure 2**) is as follows: locate the patient’s sex on the sex axis. Draw a line straight upward to the point axis to establish how many points toward the probability of CLNM the patient may get. Repeat the process for each of the other variables. Calculate total points for each of the predictors. Pinpoint the final score on the total point axis. Draw a line straight down to determine the patient’s predicted probability of CLNM. For example, the nomogram predicted a PTC male (33 points) patient with the tumor more than 0.5cm (34 points). The tumor had the ETE (100 points). The total point was 167 for this patient. This patient had more than 80.0% chance of CLNM.

**Figure 3:**
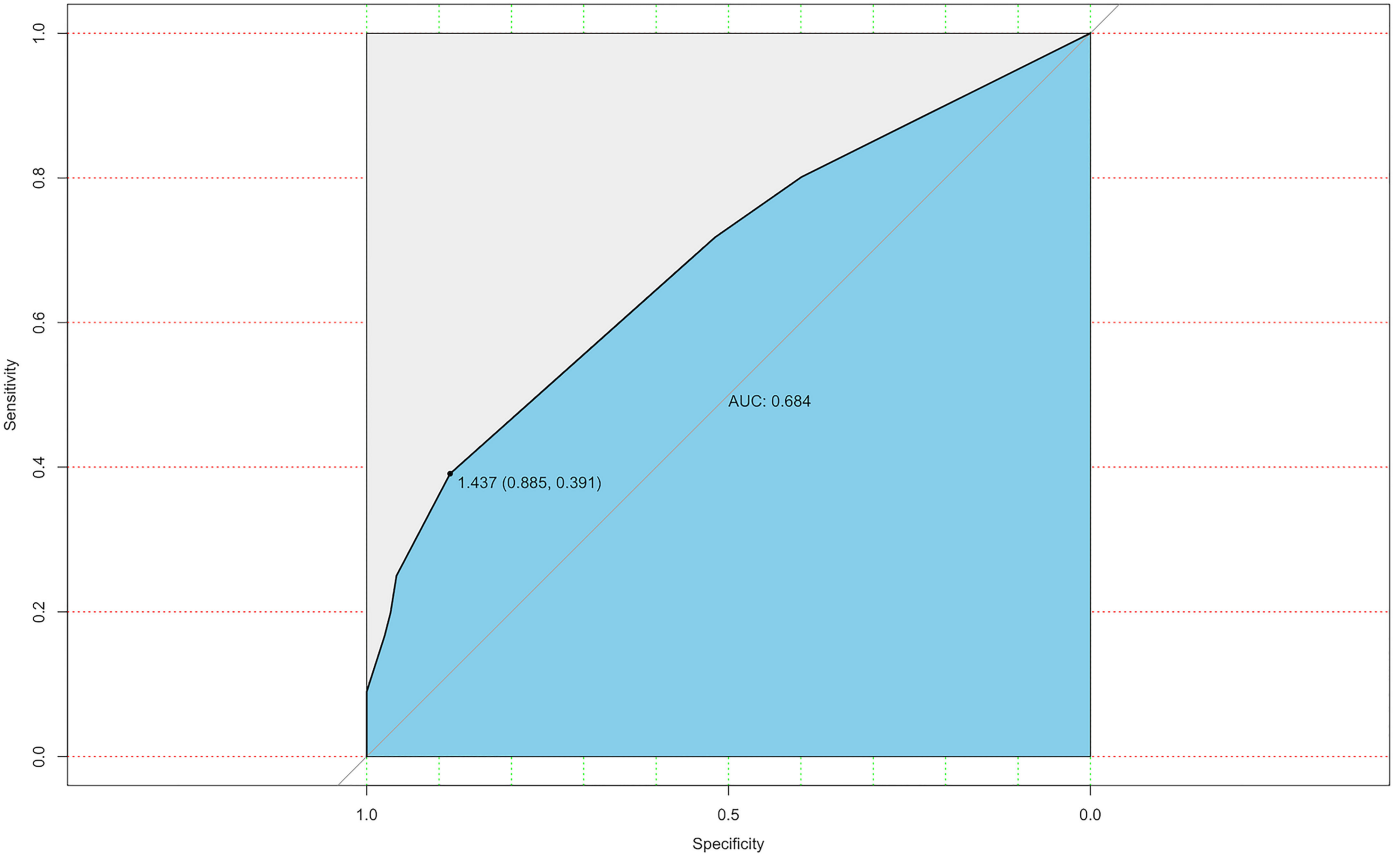
ROC curve for predicting model.

**Figure 4:**
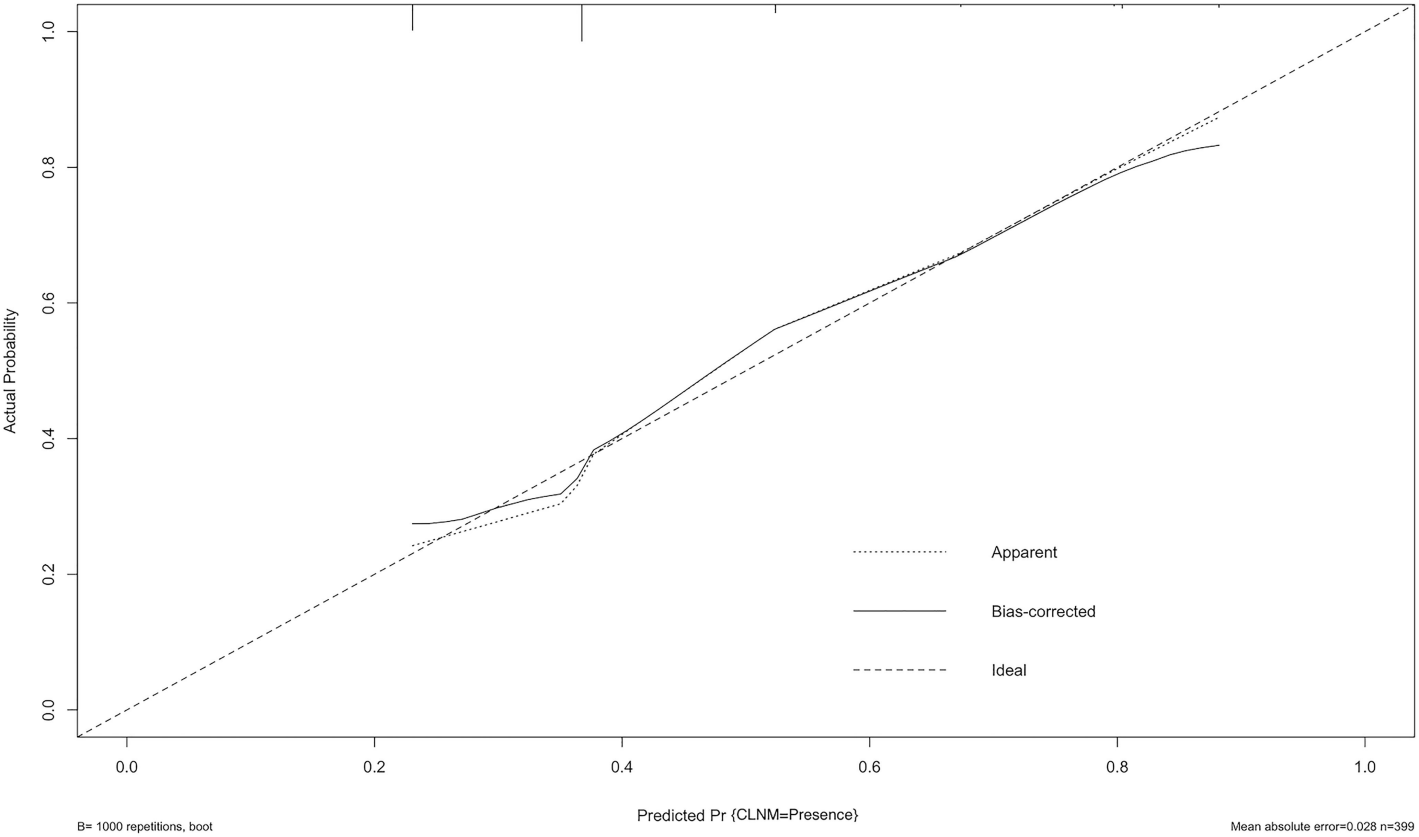
Calibration curve of the model. The diagonal dashed line represents the ideal prediction by the perfect nomogram; the solid line represents the calibration estimate from internally validated model; the dotted line indicates the apparent predictive accuracy. The closer the solid line is to the dotted line, the stronger the predictive ability of the model.

The ROC curve was also constructed, and an area under curves (AUC) of 68.4% (95% CI: 0.635-0.774) with specificity and sensitivity as 0.885 and 0.391 respectively showed good calibration (**Figure 3**). Additionally, the optimal cut-off value of the nomogram was 1.437, which was considered good in the differential diagnosis of CLNM. The calibration chart graphically showed an excellent agreement between the actual probability of CLNM and the metastasis probability predicted by the nomogram, with a mean absolute error of 0.028 (**Figure 4**).

### Risk Factors for RFS

**Figure 5:**
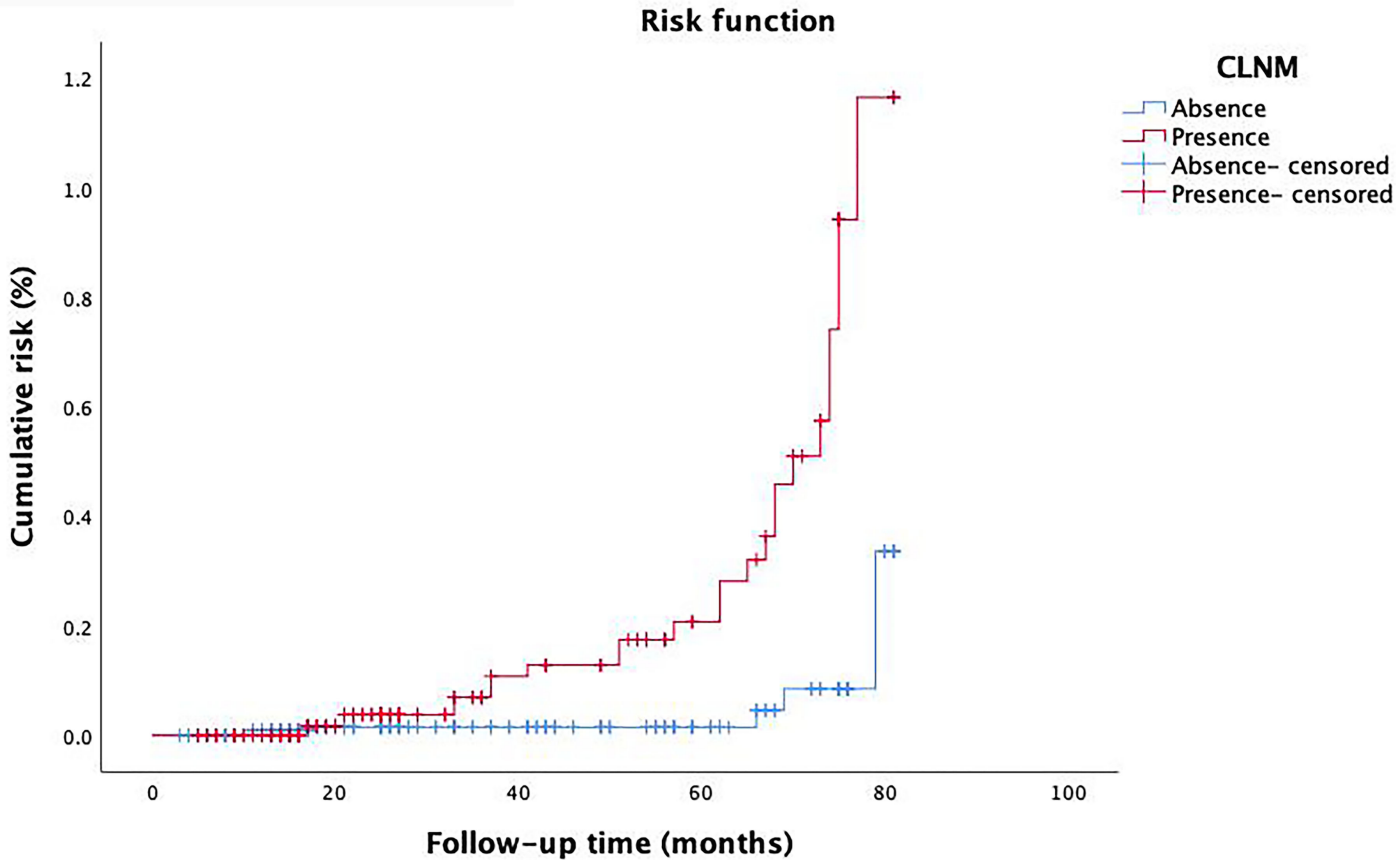
The cumulative risk in patients with CLNM and patients without CLNM.

We performed univariate analysis related to RFS to identify variables that affect the risk of recurrence (**Table 2**). Risk factors such as tumor size, multifocality, ETE, vascular invasion, and CLNM showed statistically significance associated with RFS (P=0.003, P=0.024, P=0.015, P=0.003, P=0.005, respectively). However, RFS was not significantly related to other factors studied. The risk of recurrence was 3.700, 8.831, 2.934, and 4.340 times higher in patients with tumor size > 0.5 cm, multifocality, ETE and vascular invasion, respectively. It was also found that patients with CLNM had a 3.730 times higher risk of recurrence than that of whom without CLNM. Furthermore, with the extension of follow-up time, the cumulative risk of CLNM patients was substantially higher than that of whom without CLNM (*P*<0.001) (**Figure 5**).

**Table 2:** Cox proportional hazards model demonstrating factors associated with recurrence-free survival in PTMC patients.

Characteristics	HR	95% CI	*P* value	Cumulative risk
Age (Y)				
≥55	1			
<55	1.601	0.654-3.924	0.303	
Sex				
Female	1			
Male	1.474	0.549-3.959	0.442	
Tumor size (cm)				
≤0.5	1			
>0.5	3.700	1.543-8.875	0.003	
Multifocality				
Absence	1			
Presence	3.111	1.165-8.306	0.024	
ETE				
Absence	1			
Presence	2.934	1.238-6.955	0.015	
Vascular invasion				
Absence	1			
Presence	4.340	1.648-11.432	0.003	
CLNM				
Absence	1			
Presence	3.730	1.482-9.388	0.005	<0.001

Y, year; SD, standard deviation; ETE, extrathyroidal extension; CLNM, central lymph node metastasis.

## Discussion

The prevalence of PTMC has increased rapidly in the past few years thanks to the development and application of US and necessary FNAB (15). According to Chinese Guidelines for Diagnosis and Treatment of Differentiated Thyroid Cancer (2012), lobectomy and prophylactic CLND was recommended for patients with tumors <1 cm in size. However, previous studies had shown that preventive CLND does not significantly benefit patients with low-risk PTC (16, 17). It was suggested that active surveillance rather than surgery could be taken for low-risk PTMC by the guidelines of the American Thyroid Association (2015) (2). However, some previously considered low-risk PTMCs still progressed during surveillance (18, 19). Therefore, surgical treatment options are more often taken by patients diagnosed with PTMC in China (20–22). Nevertheless, considering the uncertain efficacy and high risk of complications, it was still controversial whether to perform preventive CLND while no obvious evidence of CLNM was found before surgery (23). Thus, careful assessment of cervical lymph node status and determination of risk factors related to CLNM before surgery could guide PTMC patients to adopt appropriate surgical methods and avoid unnecessary complications (24).

According to our study, a logistic regression model based on US and clinical variables was set up to predict the probability of CLNM in patients with PTMC. Factors associated with CLNM of PTMC patients were studied in depth through univariate and multivariate analyses. As previously reported, the occurrence rate of CLNM confirmed by pathologists was between 29.3% and 60.9% in cN0 PTC patients (25–28). In present study, the occurrence rate of CLNM was 39.1%, which was in accordance with the results of previous studies. The incidence of CLNM was higher in patients of male, age <55 years, tumor size >0.5 cm, ETE, and vascular invasion respectively according to our research. Further, multivariate analysis revealed that male, tumor size and ETE were independent risk factors for CLNM. These were similar to the results of some previous studies (29, 30). In addition, tumor size, multifocality, ETE, vascular invasion, and CLNM were found to be significantly correlated with RFS in patients with PTMC, and the cumulative hazard of CLNM patients was closely related to the postoperative follow-up time. Therefore, we urgently need a prediction system for individual CLNM probability to determine the extent of surgery and ultimately improve the prognosis.

To further predict the probability of CLNM, our study utilized the previous variables based on the multivariate analysis to construct a nomogram system (**Figure 2**). According to the point scale, we assigned a score to each variable. Next, the CLNM risk for each subject was determined by adding up all the total scores and identifying them on the total point scale. The nomogram incorporates all related clinical and US features to provide an individual risk assessment of CLNM preoperatively, which may avoid unnecessary expansion of extent of surgery. The AUC of the nomogram in our study was 0.684 (95%CI, 0.635-0.774), suggesting good discrimination. Surgeons could make individualized treatment plans accordingly, which is in conformity with the present tendency of personalized precision healthcare (31–34).

Our research still has some potential limitations which we hope to address in the following research. First, this was a retrospective observational research. Biases and errors in retrospective studies are often higher than in prospective studies (35). Hence, the prediction performance may be affected by the retrospective design of the study. Second, although we gathered cases from two hospitals, the sample size and the number of variables studied was not sufficiently large. Therefore, future studies involving more sample sizes and variables are needed, and further multicenter external validation are also needed to develop high-level evidence. More latent variables that are significant for CLNM prediction may be discovered, which can make the nomogram more complete. Third, the performance of the nomogram may be partially affected by different US instruments and operators with different experience. Because of the limitations of US, occult metastasis may not be detected during follow-up and cumulative risk calculation may also be affected (10, 36). Furthermore, we did not discuss whether prophylactic CLND in high-risk patients would reduce the risk of recurrence and improve patient outcomes. We plan to conduct prospective studies in the future, including setting up a comparison group of patients who will not receive prophylactic CLND to explore the impact of prophylactic CLND on postoperative complications and recurrence.

In summary, sex, tumor size, and ETE were significantly related to CLNM of PTMC in our research. By using above variables, we were able to construct a nomogram to evaluate CLNM risk of PTMC and provide a reference for clinicians to make individualized treatment decisions. For patients with high-risk factors for RFS, active surgical strategies are especially important.

## Data Availability Statement

The raw data supporting the conclusions of this article will be made available by the authors, without undue reservation.

## Ethics Statement

Written informed consent was obtained from the individual(s) for the publication of any potentially identifiable images or data included in this article.

## Author Contributions

JY and J-WF: Writing - Original Draft, Software, Data Curation. W-XW and L-ZH: Validation, Formal analysis, Data Curation. JH: Conceptualization. W-HS and A-CQ: Validation, Investigation. YJ: Writing - Review & Editing, Visualization, Supervision. All authors contributed to the article and approved the submitted version.

## Conflict of Interest

The authors declare that the research was conducted in the absence of any commercial or financial relationships that could be construed as a potential conflict of interest.

## Publisher’s Note

All claims expressed in this article are solely those of the authors and do not necessarily represent those of their affiliated organizations, or those of the publisher, the editors and the reviewers. Any product that may be evaluated in this article, or claim that may be made by its manufacturer, is not guaranteed or endorsed by the publisher.
